# Observation and tunability of room temperature photoluminescence of GaAs/GaInAs core-multiple-quantum-well shell nanowire structure grown on Si (100) by molecular beam epitaxy

**DOI:** 10.1186/1556-276X-9-626

**Published:** 2014-11-22

**Authors:** Kwang Wook Park, Chang Young Park, Sooraj Ravindran, Ja-Soon Jang, Yong-Ryun Jo, Bong-Joong Kim, Yong Tak Lee

**Affiliations:** 1 School of Information and Communications, Gwangju Institute of Science and Technology, 123 Cheomdangwagi-ro, Buk-gu, Gwangju 500-712, Republic of Korea; 2 Current address: Samsung Advanced Institute of Technology, 130 Samsung-ro, Yeongtong-gu, Suwon, Gyeonggi-do 443-803, Republic of Korea; 3 School of Electrical Engineering and Computer Science, Department of Electronics, Yeungnam University, 280 Daehak-ro, Gyeongsan, Gyeongsangbuk-do 712-749, Republic of Korea; 4 School of Materials Science and Engineering, Gwangju Institute of Science and Technology, 123 Cheomdangwagi-ro, Buk-gu, Gwangju 500-712, Republic of Korea; 5 Advanced Photonics Research Institute, Gwangju Institute of Science and Technology, 123 Cheomdangwagi-ro, Buk-gu, Gwangju 500-712, Republic of Korea

**Keywords:** Core-shell nanowire, GaAs/GaInAs multiple-quantum-well, Molecular beam epitaxy

## Abstract

We report the observation of room temperature photoluminescence (PL) emission from GaAs/GaInAs core-multiple-quantum-well (MQW) shell nanowires (NWs) surrounded by AlGaAs grown by molecular beam epitaxy (MBE) using a self-catalyzed technique. PL spectra of the sample show two PL peaks, originating from the GaAs core NWs and the GaInAs MQW shells. The PL peak from the shell structure red-shifts with increasing well width, and the peak position can be tuned by adjusting the width of the MQW shell. The GaAs/GaInAs core-MQW shell NW surrounded by AlGaAs also shows an enhanced PL intensity due to the improved carrier confinement owing to the presence of an AlGaAs clad layer. The inclined growth of the GaAs NWs produces a core-MQW shell structure having a different PL peak position than that of planar QWs. The PL emission by MQW shell and the ability to tune the PL peak position by varying the shell width make such core-shell NWs highly attractive for realizing next generation ultrasmall light sources and other optoelectronics devices.

**PACS:**

81.07.Gf; 81.15.Hi; 78.55.Cr

## Background

Since the first demonstration by Wagner and Ellis [1], nanowires (NWs) have attracted extensive attention and have found application in solar cells [2], nano-lasers [3], and light-emitting devices [4]. The increasing research attention towards NWs stems from their interesting properties, such as low dimensionality and high surface-to-volume ratio [5]. It is also possible to integrate lattice-mismatched materials during NW formation [6] a feature which paves the way to fabricating optoelectronic devices with improved device performance. The possible wavelength range of operation of NWs is decided by the band gap of its constituent material, which therefore restricts the use of homogeneous single material NWs. On the other hand, heterogeneous NWs made of a core material encapsulated by a shell (also known as core-shell NWs) can operate at a broader wavelength range since they are comprised of materials with different band gaps. Furthermore, surface states which are detrimental to photovoltaic applications, present in homogeneous NWs, can be alleviated by the presence of a shell material in core-shell NWs. The shell material can also act as a gain medium for light-emitting applications. All these features make core-shell NWs highly attractive for optoelectronic device applications, and hence, core-shell NWs have been extensively studied in various material systems such as Ge-Si [7], nitride [3,8], arsenide [9,10], phosphide [11,12], antimonide [13] based compound material systems, and so on.

Until now, various growth methods such as metal-organic chemical-vapor deposition (MOCVD) [14], molecular beam epitaxy (MBE) [9,10,15], chemical beam epitaxy (CBE) [16], chemical vapor deposition (CVD) [17], and laser ablation [18], and various materials such as GaInAs, AlInAs, and GaAs have been used to realize homogeneous NW and their epitaxial alloys to form heterogeneous NWs. Among the various growth methods, MBE stands out due to its ability to provide nanostructures having high crystalline quality and sharp atomic interfaces. Of the various materials, GaInAs and GaAs are extremely attractive for making various optoelectronic devices due to their high electron mobility, low effective mass, and the ability to generate higher photocurrent, as well as for photonic devices due to their direct band gap and high-gain and -absorption coefficients. Heterogeneous NWs formed of an epitaxial alloy of these materials would therefore be promising for realizing future electronic/photonic devices with enhanced properties and novel applications.

Despite the technological importance of GaInAs, there are very few reports in the literature describing the growth and characterization of GaInAs NWs. This is due to the fact that GaInAs NW growth is complicated: the Ga and In elements have different growth behaviors, such as solubility, diffusion properties, source decomposition efficiencies, and so on [19,20]. Overcoming these obstacles would pave the way for the rapid development and deployment of GaInAs-based nanowire electronic/photonic devices.

Recently, the MOCVD growth of GaAs/GaInAs core-shell nano-needle structures was reported [21] although the study mainly focused on structural analysis, and little attention was given to studying their optical properties at room temperature. Understanding the optical properties of NWs, particularly at room temperature, is crucial for designing NW-based optoelectronic devices, since practical NW-based devices are expected to operate at room temperature. Moreover, multiple shell NWs studied by previous researchers were grown using gold-catalyst [22] which unfortunately introduces deep-level carrier traps [23,24] degrading device performance. This limits their usage for optoelectronic device applications or requires silicon dioxide pre-deposition prior to epitaxial growth, involving additional process steps and complicating the NW formation. Since the first demonstration of catalyst-free selective-area MBE growth of GaAs/AlGaAs core-shell NWs on substrates pre-deposited with a thin layer of silicon dioxide by Morral et al. [10] in 2008, several researchers over the last few years have grown the simple core-shell [9,25,26] to the complex core-shell structure including quantum dot embedded nanowire [27,28] as well as InAs nanotubes [29] formed by selective etching of GaAs/InAs core-shell NWs; however, the optical and structural analysis on the GaInAs-related multiple core-shell NWs has not been reported yet.

In this work, we report the room temperature photoluminescence (PL) emission and the room temperature optical characterization of MBE-grown GaAs/GaInAs core-multiple-quantum-well (MQW) shell NWs. GaAs/GaInAs MQW shells having different well widths surrounded by AlGaAs clads were grown on self-catalyzed GaAs NW cores without using any gold catalyst or requiring any pre-deposition of oxide materials or further post-processing. We demonstrate the growth of GaAs NW core on (100) silicon substrate, and the optical characterization of GaInAs MQWs with various quantum-well widths grown on the GaAs NW core. The samples were structurally characterized by conventional transmission electron microscopy (TEM), scanning transmission electron microscopy (STEM), field emission scanning electron microscopy (FE-SEM), and energy dispersive X-ray (EDX) spectrometer measurements. Cross-sections of the grown NWs were analyzed by using cross-sectional TEM measurements which revealed the formation of GaInAs quantum-well layers, GaAs barriers, and AlGaAs clad layers. For optical characterization of the grown NWs, PL measurement system (Nanometrics RPM2000) was used. We find that the PL peak position of GaAs/GaInAs core-MQW shell NWs can be tuned by changing the GaInAs shell width, and the presence of an AlGaAs layer assists in enhanced carrier confinement leading to room temperature PL emission.

## Methods

Samples studied in this work were grown on p-type (100) silicon substrate using VG V80H-10 K MBE system equipped with a valved cracker effusion cell for arsenic dimer (As_2_) source. For group-III elemental sources, conventional K-cells with linear motion shutters were employed. Growth temperature was measured by an infrared pyrometer which can measure temperature over a range of 250°C ~ 2000°C with an accuracy of ±0.3%. Prior to GaInAs MQW shell growth, GaAs NW cores were grown which acted as the base for depositing the MQW shell structure. For the GaAs NW growth, native oxide on the epi-ready (100) silicon substrate was thermally cleaned at 650°C for 10 min, followed by Ga droplet deposition for 1 min at 650°C without As_2_. The amount of Ga molecular beam flux corresponded to a growth rate of 2.27 Å/s. Then, GaAs NWs were grown immediately while the MBE growth chamber pressure was kept at 8.0 × 10^-7^ mbar for 1 h until the NW growth was finished. Throughout the growth, the substrate was rotated at 9 rpm, and the V/III ratio was kept around 8 to initiate self-catalyzed growth of nanowires [30]. To investigate the effect of growth temperature on GaAs NW formation, growth temperature was varied from 600°C to 670°C.

**Figure 1:**
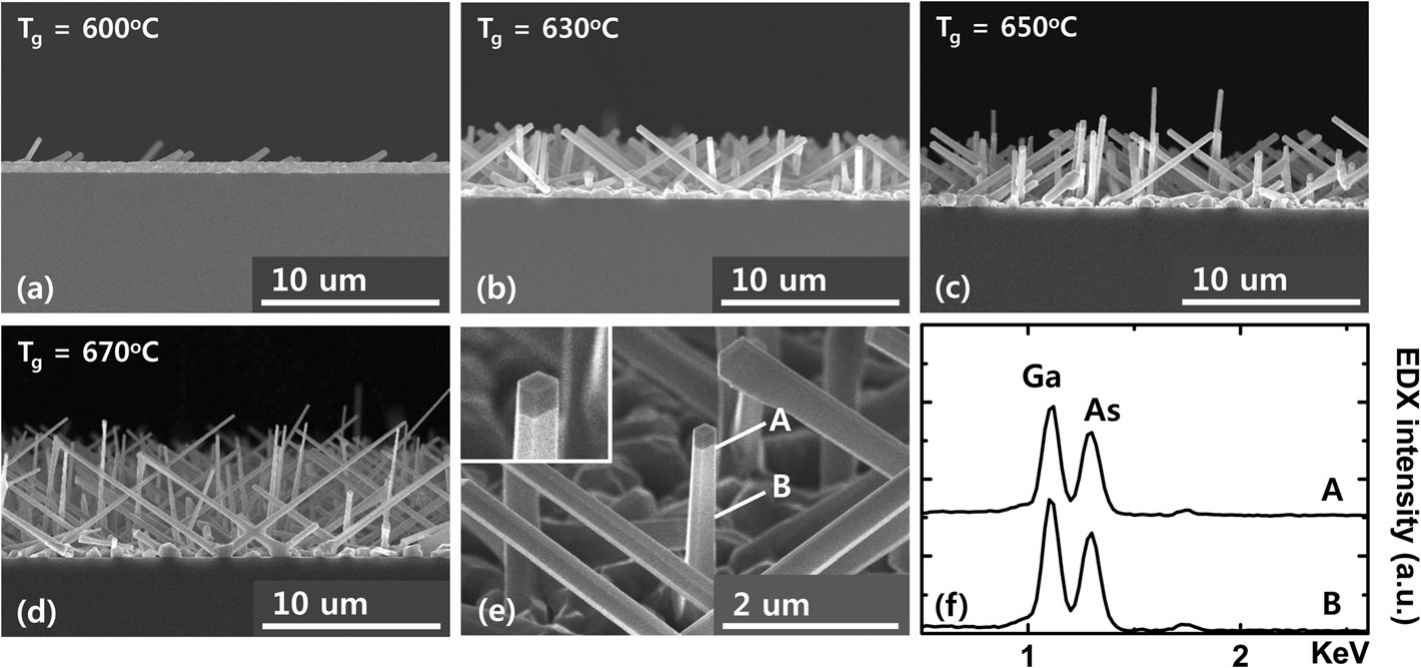
**SEM images of GaAs NWs on (100) silicon substrate with different growth temperatures. (a)** 600°C, **(b)** 630°C, **(c)** 650°C, **(d)** 670°C, **(e)** close-up view of a GaAs NW core, and **(f)** EDX spectra taken at the middle [B] and top [A] of a GaAs NW core (see Figure 1e). The inset of Figure 1e shows the clear formation of a hexagonally shaped NW.

Figure 1a,b,c,d,e shows the SEM images of the GaAs NWs grown at different temperatures. As shown in Figure 1, most of the GaAs NWs grown on (100) silicon substrate are oriented at an angle of 35° with respect to the substrate surface, indicating that the wires follow the lattice orientation of the substrate. As the growth temperature increases from 600°C to 670°C, the density of the NW as well as the length of individual NWs increases, as can be seen from Figure 1a,b,c,d. Furthermore, with the increase in growth temperature from 600°C to 630°C, it was found that the diameter of the NWs increases from 140 to 590 nm and then decreases to 170 nm at a growth temperature of 670°C, and a similar behavior has been reported in ref. [31]. On the other hand, with increasing growth temperature, the NW length increases from 1.3 to 13.3 μm. Considering the requirements that the NW core should be rigid and long enough so as to be used for depositing the shell material, 650°C was chosen as the optimum growth temperature for forming the GaAs core. To investigate the constituent elements of the grown NW, EDX studies were carried out. The EDX spectra shown in Figure 1f, taken from the top and middle point of the grown NW, reveal that it consists of Ga and As elements, and the relatively similar amplitude of Ga and As peaks from the two different points indicate that the composition of the grown NW is similar along its axis. The cross-section of the grown nanowire was hexagonal [32] as shown in the inset of Figure 1e having a mixture of wurtzite (WZ) and zinc blende (ZB) structures. This is due to the comparable free energies of WZ and ZB structures as the diameter of the grown nanowire exceeds beyond the critical diameter (approximately 10 to 30 nm) [33]. It is noteworthy that the cross-section of the stacking-fault-free ZB GaAs nanowire was square in shape [33].

**Figure 2:**
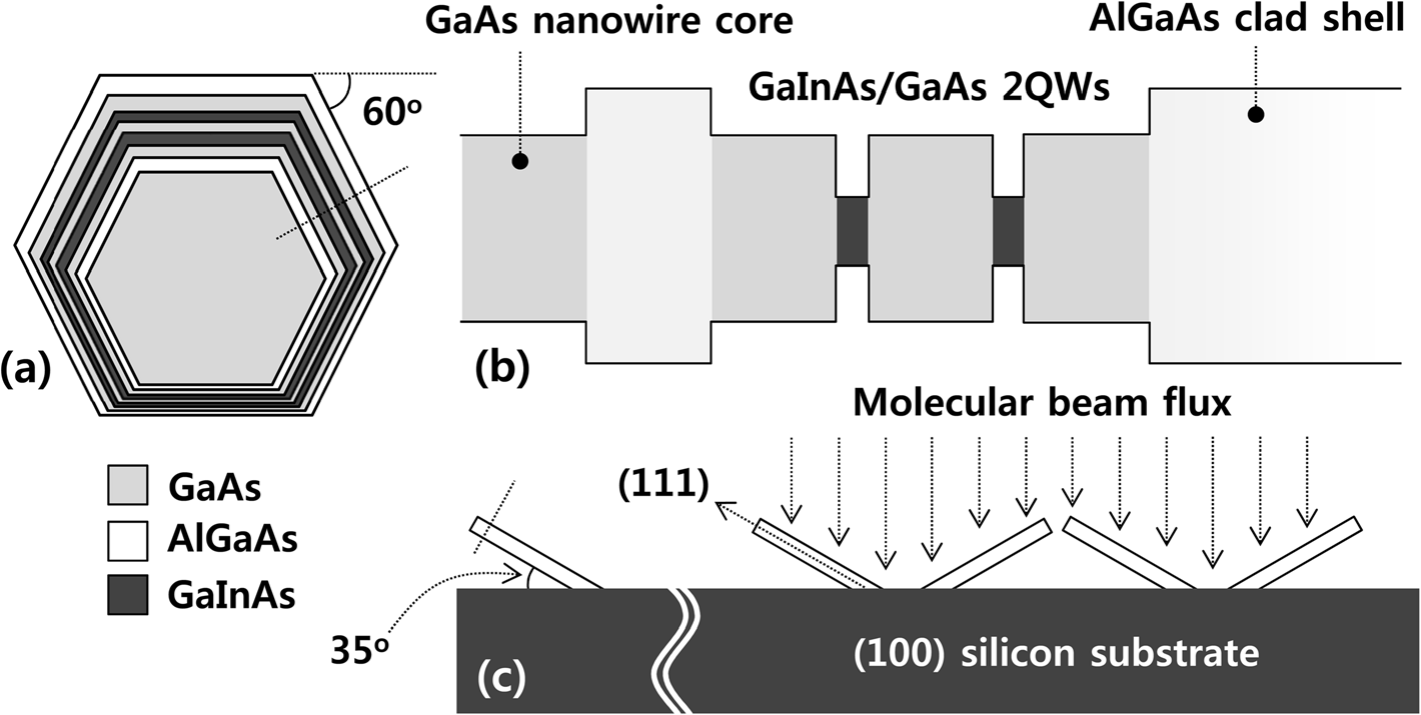
**Cross-sectional schematic of the NW to be grown, band diagram, and the inclined NW growth. (a)** Cross-sectional schematic of the NW to be grown. The center region represents the core made of GaAs surrounded by GaInAs shell with AlGaAs layer as the outermost clad layer, **(b)** band diagram of the NW along the dotted line of Figure 2a, and **(c)** schematic showing the inclined NW growth.

After completing the growth of GaAs NW core, the MBE chamber pressure and growth temperature were immediately changed to grow GaInAs MQWs. As stated before, the previously grown GaAs NWs are used as the base to grow the GaInAs MQW shell structure. The schematic of the desired core-shell structure is depicted in Figure 2. The MQW shell structure to be grown consists of two Ga_0.84_In_0.16_As quantum wells sandwiched between three GaAs barriers surrounded by an Al_0.30_Ga_0.70_As cladding layer. The conditions for growing the shell structure were the same as that for growing conventional planar GaInAs MQWs. The growth temperature and MBE background chamber pressure were kept at 570°C and 1.2 × 10^-6^ mbar, respectively, during growth. The growth temperature was carefully chosen to ensure the incorporation of desired In composition while maintaining high quality of the compositing layers [20]. The V/III ratio was kept around 12 to prevent any axial growth [16]. The GaInAs MQW shell width were 8, 11, and 16 nm, respectively, based on planar growth mode. The barrier between the individual wells was thick enough to avoid any coupling between the QWs. Planar MQWs having a thickness of 8 nm with the same structure and compositions were also grown on (100) GaAs substrate in order to compare the results obtained from the shell structure.

## Results and discussion

Prior to the structural and compositional investigation of the complete GaAs/GaInAs core-MQW shell NWs surrounded by AlGaAs layer, the compositional and structural properties of the constituent AlGaAs and GaInAs shells were investigated by TEM. To prepare the sample for TEM measurements, the cleaved as-grown samples were dipped in isopropyl alcohol (IPA) and sonicated for 5 to 10 s in order to separate the nanowires from the substrate. The droplet that contains the mixture of nanowires and IPA was dropped on the carbon film covered copper grid followed by drying of residual IPA to obtain nanowires suitable for TEM evaluation.

**Figure 3:**
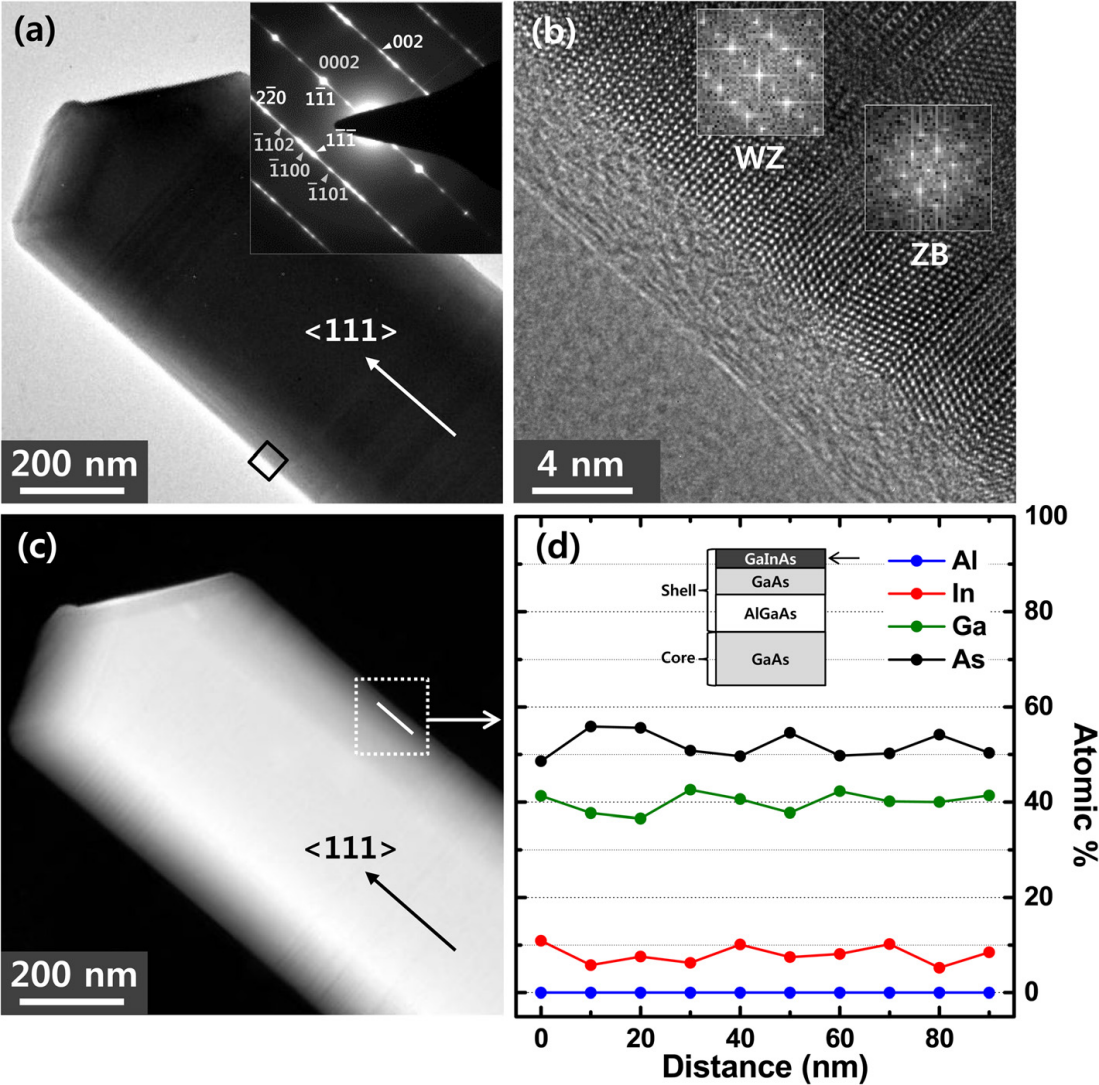
**BF TEM, HRTEM, and HAADF STEM images of GaAs/GaInAs core-shell nanowire and spot EDX data. (a)** BF TEM image of a GaAs/GaInAs core-shell nanowire (GaInAs/GaAs/AlGaAs on GaAs core NW) and **(b)** HRTEM image of the GaInAs layer from the boxed area of the nanowire in Figure 3a. The insets are the FFT patterns from the ZB and WZ structures. **(c)** HAADF STEM image of the same area of the nanowire shown in Figure 3a. The white line shown in the box represents the region along which the spot EDX was measured. **(d)** Spot EDX data revealing the atomic weight percentage of the constituent elements of the GaInAs layer of the nanowire shown in Figure 3c. Inset shows the cross-sectional schematic of the nanowire structure shown in Figure 3a. The arrow shown in the inset indicates the layer from which spot EDX is taken.

Figure 3a shows the bright field (BF) TEM image of the core-shell nanowire grown without the AlGaAs clad layer. The cross-sectional schematic of the nanowire structure is described in the inset of Figure 3d. For this sample, the GaInAs well layer and one of the GaAs barrier layer is present, while the second GaAs barrier layer and the outermost AlGaAs clad layer are absent. The diameter of the nanowire was approximately 450 nm, and the expected width of GaInAs well was 16 nm. The selected area diffraction (SAD) pattern in the inset of Figure 3a indicates that the grown nanowire is crystalline having a mixture of ZB and WZ crystalline structures due to the comparable-free energies of WZ and ZB in large diameter NWs [33]. The stacking faults located between the two structures give rise to the streaks shown in the SAD pattern. By comparing the diffraction pattern with the BF image, we find that the nanowire is grown along [111] of the ZB structure which is equivalent with [0001] in the WZ structure. Figure 3b shows the high-resolution transmission electron microscopy (HRTEM) image of the GaInAs layer from the boxed area of the nanowire shown in Figure 3a, and the fast Fourier transform (FFT) images as insets in Figure 3b. From these, we can identify the two different nanowire sections, i.e., ZB and WZ structures, which are confirmed by the FFT patterns of the corresponding areas. Moreover, we note that the FFTs of the outermost layer correspond to the diffraction pattern of the entire layers shown in Figure 3a, indicating that the shell layer is deposited epitaxially on the core nanowire. Figure 3c shows the high-angle annular dark field scanning transmission electron microscopy (HAADF) STEM image of the boxed area of the nanowire shown in Figure 3a. HAADF STEM image was used for composition analysis over TEM image due to the former’s higher sensitivity to the variation in the atomic number of atoms and/or relative differences in the electron density distribution of the samples. Spot EDX measurements were carried out to determine the constituent elements as well as the composition of the GaInAs well layers.

**Figure 4:**
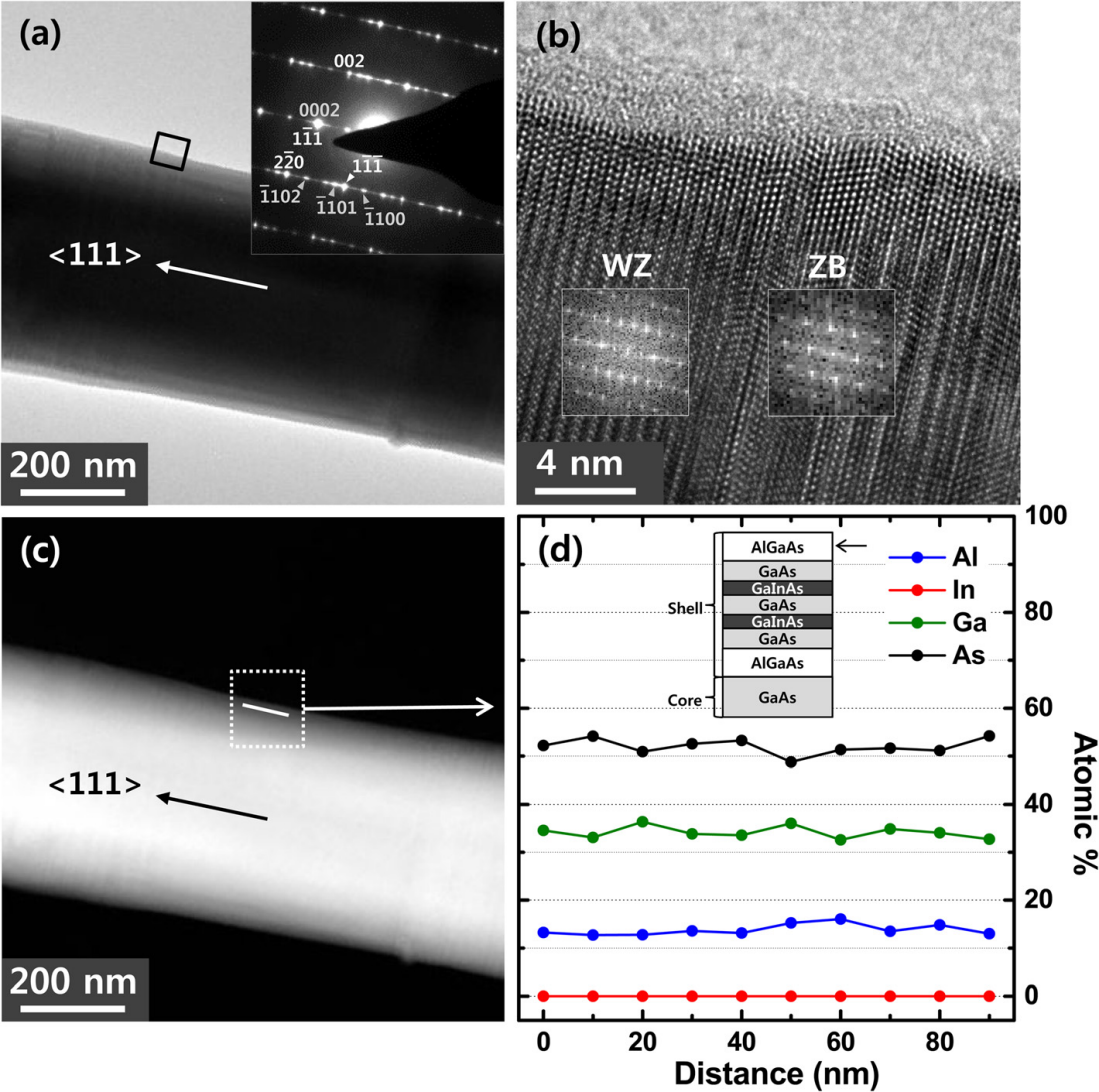
**BF TEM, HRTEM, and HAADF STEM images of a nanowire and spot EDX data. (a)** BF TEM image of a nanowire with the complete core-MQW shell structure including the outermost AlGaAs layer. **(b)** HRTEM image of the boxed area in Figure 4a, and the insets show the corresponding FFT patterns from ZB and WZ structures. **(c)** HAADF STEM image of the same nanowire shown in Figure 4a. The white line shown in the box represents the region along which the spot EDX was measured. **(d)** Spot EDX data revealing the atomic weight percentage of the constituent elements of the outermost AlGaAs layer in the nanowire shown in Figure 4c. The inset shows the cross-sectional schematic of the nanowire structure shown in Figure 4a. The arrow shown in the inset indicates the layer from which spot EDX is taken.

Figure 3d shows the spot EDX data taken from ten different points on the GaInAs layer that was situated at a distance of 10 nm away from the nanowire edge. The spacing between each successive point was also 10 nm. Note that the point resolution of the EDX is approximately 1 nm. The average atomic composition of In is 8.0% with a range of deviation of approximately 2.9%, while the average atomic compositions for Ga and As are 39.5% and 53.8% with the range of error of approximately 3.4% and approximately 3.9%, respectively. These values are close to the predicted composition of Ga_0.84_In_0.16_As thin film. It is also evident from Figure 3d that the spot EDX from the GaInAs well layer reveals the presence of In, Ga, and As elements, while Al is absent, and hence, no signal corresponding to Al is observed in Figure 3d. The EDX analyses thus confirm the presence of GaInAs in the grown MQW structure, though we are not able to measure the exact thicknesses of the GaInAs and GaAs layers.Figure 4a shows the BF TEM image of a nanowire with the complete core-shell structure including AlGaAs as the outermost layer. To construct this nanowire structure, we added GaAs, GaInAs, GaAs, and AlGaAs layers to the nanowire shown in Figure 3a. The schematic of the core-shell nanowire having all the layers is shown in the inset of Figure 4d. The SAD pattern of the entire nanowire in the inset of Figure 4a is identical to that shown in the inset of Figure 3a, indicating that the outer shells are grown epitaxially on the wire shown in Figure 3a. This is confirmed by the HRTEM image (Figure 4b) and the corresponding FFT images (inset of Figure 4b) of the layer including the AlGaAs outermost layer, demonstrating the presence of both ZB and WZ structures in the layer.

The constituent elements of the outermost AlGaAs shell were investigated using high-resolution spot EDX measurements and are shown in Figure 4d. The measurements were carried out from ten different locations of the AlGaAs layer that was situated at a distance of 10 nm away from the nanowire edge. The interspacing between each point was also 10 nm. In the case of Al, the average atomic composition is 13.8% with the error range of 2.7%, while the average atomic compositions for Ga and As are 34.2% and 52% with the error ranges of 2.2% and 3.3%, respectively. These values are in agreement with the composition measured from Al_0.30_Ga_0.70_As thin film. As seen from Figure 4d, the measured data reveals the presence of Al, Ga, and As elements while In is absent. The absence of In is not surprising because the outermost layer of the NW is made of AlGaAs layer and hence, In should not be detected in EDX scan. This confirms that the outermost shell of the grown nanowire is indeed an AlGaAs layer; its role is to enhance the carrier confinement.

To further investigate the structural details and to confirm that the growth of the complete structure of GaAs/GaInAs MQW shell surrounded by AlGaAs clad layer was achieved, cross-sectional TEM analysis of the complete NW structure was performed. The sample preparation for obtaining cross-sectional TEM image was done as follows. The sample having nanowires was sonicated in order to detach the nanowires from the mother substrate. The detached nanowires were then scattered on the carrier substrate, and one of them was selected by SEM for performing cross-sectional TEM measurement. The selected nanowire was then thinned by focused ion beam (FIB) milling, and the cross-sectional image was obtained using TEM.

**Figure 5:**
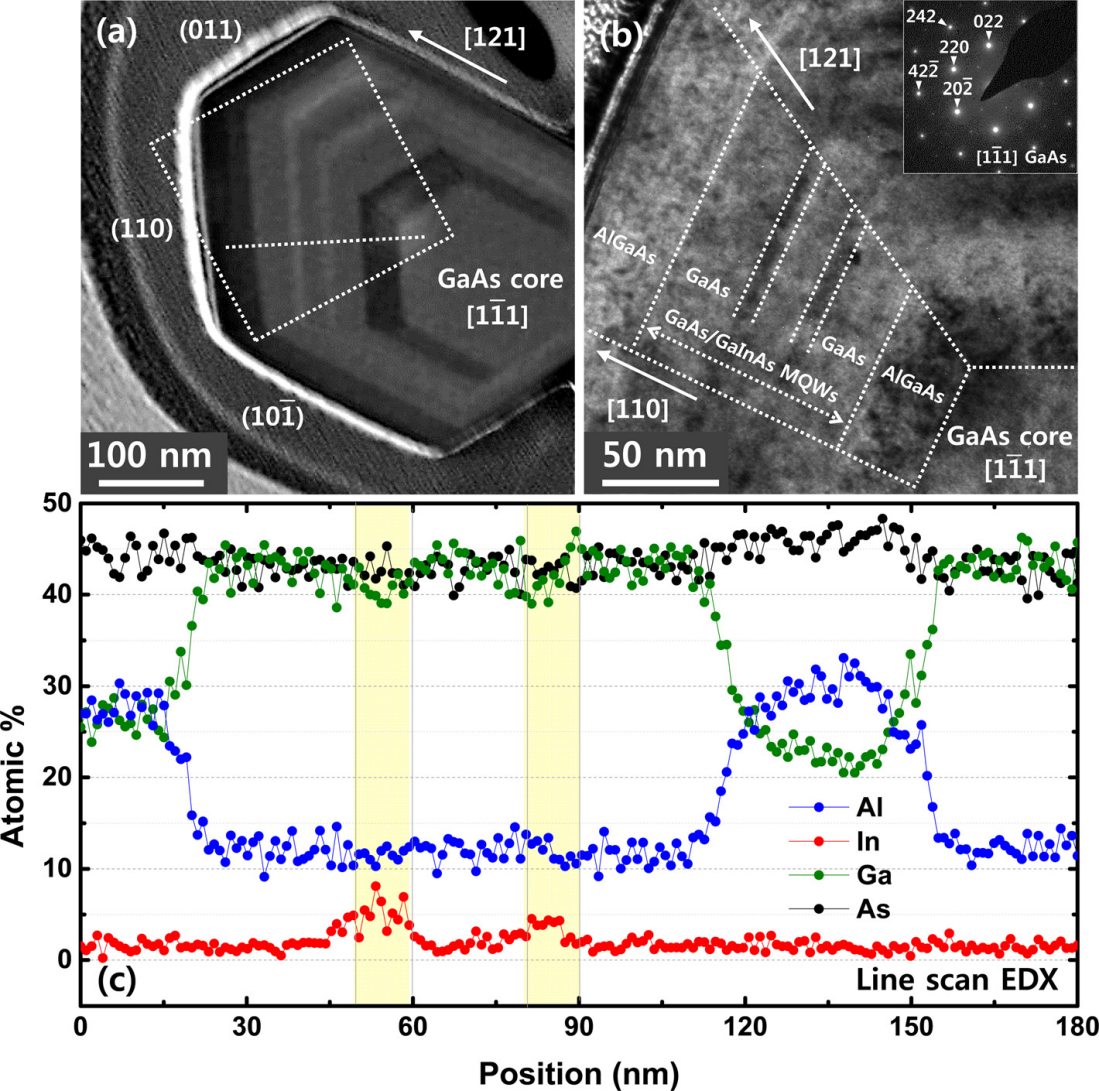
**Cross-sectional HAADF-STEM image, close-view of the cross-section BF TEM, and line scan EDX profile. (a)** Cross-sectional HAADF STEM image of the grown GaAs/GaInAs core-MQWs (expected GaInAs shell thickness of 16 nm) surrounded by AlGaAs clads grown on GaAs core and **(b)** close view of the cross-section BF TEM of the boxed area clearly shows multiple layers including GaAs/GaInAs MQWs and AlGaAs clads. **(c)** Line scan EDX profile (taken along the white dotted line shown inside the dotted box in Figure 5a) shows GaAs, GaInAs, and AlGaAs shells. The actual GaInAs shell thickness of the 16-nm-thick GaInAs shell was approximately 12 nm. The yellow regions have a higher In composition and represent the GaInAs well layers.

Figure 5a shows such an HAADF STEM image of a NW with the complete structure having a 16-nm-thick GaInAs shell, and the BF TEM close-up view of the white boxed region in Figure 5a is shown in Figure 5b. It can be seen that the cross-sectional image clearly reveals the two GaInAs quantum wells, where each GaInAs well is sandwiched between GaAs barriers. The innermost AlGaAs layer that surrounds the GaAs nanowire and the outermost AlGaAs layer that surrounds the shell structure can also be seen. The cross-sectional TEM measurement thus confirms the formation of GaInAs quantum-well layers, GaAs barriers, and AlGaAs clad layers. The cross-sectional image of Figure 5a also reveals that the thickness of the shell that surrounds the nanowire is not uniform. The shell that is on the facet of the nanowire which directly faces the molecular beam flux is thicker while the shell that is not directly exposed is thinner [9]. From the inset of Figure 5b, it is found that the nanowire is grown along [111] direction. The line scan EDX profile of the NW cross-section taken along the outermost AlGaAs shell to the GaAs core (the white dotted line shown inside the dotted box in Figure 5a) depicted in Figure 5c clearly reveals the presence of GaAs, GaInAs, and AlGaAs shells. The EDX line scan does not provide quantitative information of the compositing layers due to the limited spatial resolution of EDX and/or sample drift during measurement. However, via spot EDX measurement, the quantitative details of the compositing layers can be obtained, and the regions having higher In composition represent the GaInAs QW layers.

**Figure 6:**
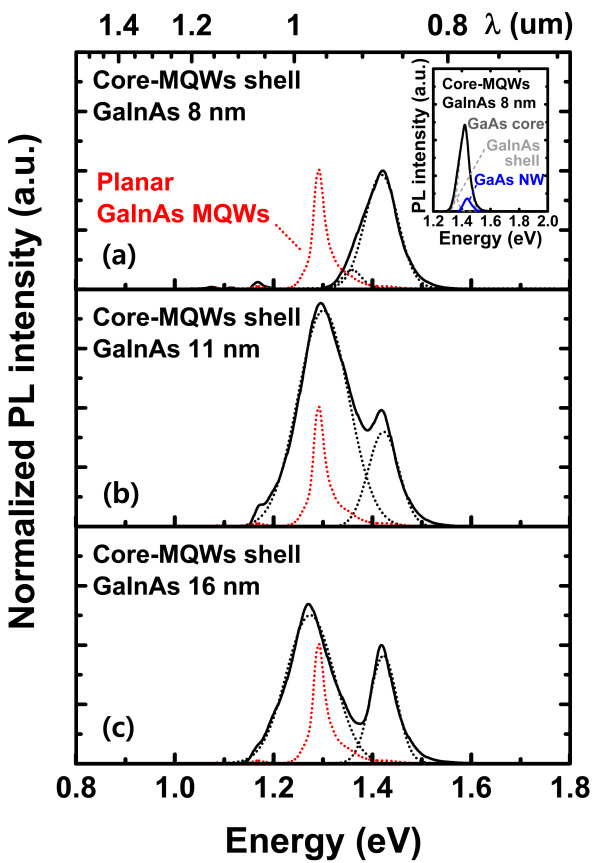
**Normalized PL spectra of GaAs/GaInAs MQW shell on GaAs NW with different quantum-well widths. (a)** 8.0 nm, **(b)** 11 nm, and **(c)** 16 nm. The red line represents the PL spectra of the planar GaInAs MQWs grown on GaAs substrate. Inset in **(a)** shows the increase of PL intensity of GaAs NW due to the presence of MQW shells. PL peak corresponding to GaInAs shell shifted to lower energy with the increase of GaInAs shell width.

Figure 6a,b,c shows the normalized PL spectra (normalized with respect to PL of GaAs core) of GaAs/GaInAs core-MQW shell NWs surrounded by AlGaAs structures with three different well widths measured at room temperature. The composition of 8-nm-thick planar GaInAs MQWs grown on GaAs substrate remained the same as that of the 8-nm-thick MQW shell NW grown on GaAs core. To the best of our knowledge, this is the first observation of room temperature PL spectra from a MBE grown GaAs/GaInAs core-MQW shell NW surrounded by AlGaAs clads. Unlike the previous report [21], the presence of the AlGaAs clad layer helps the carriers to be tightly confined in the quantum wells [34] preventing carriers escaping from the wells and enabling room temperature PL measurements. The PL spectra shown in Figure 6 are comprised of two peaks; one around 1.42 eV which corresponds to the PL emission from the GaAs NW core and the other situated at an energy lower than 1.42 eV, which corresponds to the PL emission from the GaInAs shell. The PL peak energy of the grown GaAs NW core is slightly lower than that of pure ZB GaAs NW due to the fact that the grown NWs are polytypic (i.e., a mixture of WZ and ZB structures) [35]. The inset of Figure 6a shows the comparison of the PL intensity from a bare GaAs NW and of core-MQW shell NW structure which has an 8-nm-thick GaInAs shell. The PL spectrum of the core-MQW shell NW is predominated by the emission from the GaAs core with the peak around 1.42 eV, and the PL spectrum of the bare GaAs NW is also found at around 1.42 eV. However, as seen from the figure, the PL peak intensity of GaAs NW is greatly enhanced owing to the presence of the shell structure acting as a passivation layer which suppresses non-radiative recombination centers [35].

As shown in Figure 6, when the GaInAs shell width is increased from 8 to 16 nm, there is no significant change in the position of the PL peak at around 1.42 eV (which corresponds to the PL from GaAs core), and this is due to fact that all the GaAs NW core samples were grown under the same growth conditions. However, the PL peak situated below 1.42 eV which originates from GaInAs is red-shifted from 1.35 to 1.30 eV with the increase of GaInAs shell width from 8 to 11 nm. The PL peak intensity of the 8-nm-thick GaInAs shell was smaller than the PL intensity from GaAs NW core. However, the intensity of the PL from GaInAs became larger than that from the GaAs NW core as the width of the GaInAs shell was increased from 8 to 11 nm due to an increase in the gain volume. With a further increase in the GaInAs shell width from 11 to 16 nm, the PL peak continued to red-shift from 1.30 to 1.25 eV. The PL peak intensity of the 16-nm-wide GaInAs shell, on the other hand, decreased and became comparable with the PL peak intensity of GaAs NW core. This is presumably due to the onset of plastic relaxation [36] for a shell thickness far beyond the critical layer thickness of GaInAs on GaAs [37]. The critical layer thickness of individual layers could be much larger compared to the case of planar film growth, enabling coherent growth of layers in core-shell NWs as the core diameter becomes sufficiently small [38]. Note that, in our case, the core NW diameter of the core-shell NWs is in the range of hundred nanometer scale, and hence, the concept of critical layer thickness in the planar film growth regime still holds valid [39].

The red-shift of the smaller energy peak with the increase in the GaInAs shell width can be directly explained from elementary quantum mechanics [40]. However, the PL peak positions of the GaInAs MQWs grown on GaAs substrate, and that of the GaInAs shell structure grown on GaAs NWs are not identical even though both were grown with the same sequences and conditions. The PL peak of 8-nm-thick planar GaInAs MQWs grown on GaAs substrate was around 1.27 eV; however, the PL peak of GaInAs MQWs grown on GaAs NWs with the same growth duration was around 1.35 eV, indicating an increase in band gap of approximately 80 meV due to variation in well width.

As described in the ‘Method’ section, the GaAs NW grown along the (111) direction was inclined with respect to the substrate surface. As a result, the molecular beam flux which incidents vertically on the substrate surface does not result in the growth of conventional epitaxial layers, in contrast with the growth of planar quantum wells. We presume that the incident flux is initially deposited on the NW facets that are directly exposed to the flux, and the adatoms of the incident flux then diffuse along the other facets that are not directly exposed, covering the whole NW surface. With the GaAs NW core (larger than 300 nm in diameter), the surface energy of the adatoms deposited on the NW is very close to that deposited on a planar substrate [41]. As a result, the adatoms deposited on the NW facets that are not directly exposed to the molecular beam flux can possibly form a layer that is slightly thinner than that formed at facets directly exposed to flux. Furthermore, the incident angle of the molecular beam flux was tilted at an angle of 35° with reference to the substrate surface, due to the change in the growth direction [42]. Due to the hexagonal cross-sectional geometry along with the tilted growth, the thickness of the deposited shell layer is expected to be smaller by a factor of Sin(90° - 35°) × Sin(90° - 30°) compared to that of a QW structure grown in planar mode. The spectral bandwidth of the PL emission from the GaInAs MQW shell changed from 0.04 to 0.12 eV and to 0.11 eV with the increase of the MQW shell width from 8 to 11 nm and to 16 nm, respectively. This is larger than the spectral bandwidth of the PL emission from planar GaInAs MQWs (0.03 eV). The wider and rather an irregular variation in the spectral bandwidth of the PL emission from MQW shell as compared with the planar MQWs might be, in part, attributed to the irregular shell width due to the inclined growth and poor uniformity of the NWs. On the other hand, the spectral width of the PL emission from GaAs core NW remained the same at around 0.06 eV revealing that they are grown under the same growth condition.

From this, the thicknesses of the grown quantum wells of the three shell structure samples are expected to be around 6.0, 8.5, and 12 nm, which are different from the well widths of 8, 11, and 16 nm of the planar quantum wells, respectively. As shown in Figure 5, the actual thickness of the shell which corresponding to 16 nm was around 12 nm as expected. From these values, we can observe that the obtained PL peak energies of the grown shell structures are well matched to that of thin film GaInAs quantum-well structures of the same thicknesses [43]. As an example, it is seen that the PL peak position of the GaAs/GaInAs MQW shell with a thickness of 8.5 nm is very close to that of a planar 8-nm-thick GaAs/GaInAs MQWs. These results indicate that the PL peak position of the core-MQW shell NWs can be tuned, and the PL intensity can be increased by simply changing the GaInAs shell width of the GaAs/GaInAs MQW shell structure, making them a potential building block for realizing ultrasmall light sources.

## Conclusions

In summary, we have reported the self-catalyzed growth of GaAs NWs on (100) silicon substrate and the subsequent growth of GaAs/GaInAs MQW shells surrounded by AlGaAs clad layer using MBE. The NWs were characterized using FE-SEM, EDX and room temperature PL measurements. EDX measurements confirmed the successful growth of self-catalyzed GaAs NW cores on silicon substrate, while FE-SEM measurements showed that the NW diameter increased with increasing growth temperature. GaAs/GaInAs MQW shell surrounded by AlGaAs clad was then grown on the previously grown GaAs NW core which acts as the base for growing the core-shell NW. The compositional and structural properties of the grown samples were investigated with TEM, STEM, and EDX measurements. The cross-sections of the grown NWs were analyzed by using cross-sectional TEM measurements which confirmed the formation of GaInAs quantum-well layers, GaAs barriers, and AlGaAs clad layers.

Room temperature PL was observed from the grown GaAs/GaInAs core-MQW shell NWs surrounded by AlGaAs, and the PL intensity was enhanced and broadened due to enhanced carrier confinement by the AlGaAs clad layer. Room temperature PL measurement of the core-shell structure revealed two peaks; one originated from the GaAs NW core and the other from the GaInAs MQW shells. Furthermore, the PL peak position of the GaAs core remained the same, while the PL peak of the GaInAs shell red-shifted when the GaInAs shell width was increased. In addition, PL measurements also confirmed that the well widths of a GaInAs shell grown on a GaAs core was slightly different from the expected well widths of planar quantum wells, due to the tilted growth of the GaAs NWs. The observation of photoluminescence emission at room temperature and the possibility of tailoring the room temperature PL peak by varying the GaInAs well width of core-shell NW structures makes the grown GaAs/GaInAs core-MQW shell NW an interesting and versatile candidate for realizing next generation optoelectronic devices.

## Competing interests

The authors declare that they have no competing interests.

## Authors’ contributions

KWP and CYP carried out the MBE growth and characterization of the samples. KWP and SR performed the optical analysis and characterization. JSJ, YRJ, and BJK performed the TEM measurement and structural analysis. KWP and SR drafted the manuscript. YTL supervised the whole work and finalized the manuscript. All authors read and approved the final manuscript.
